# Long-term probability of detecting drug-resistant HIV in patients starting antiretroviral therapy within the first year of HIV infection

**DOI:** 10.1186/1758-2652-13-S4-O4

**Published:** 2010-11-08

**Authors:** S Lodi, C Kücherer, AM Bakken Kran, B Masquelier, A d'Arminio Monforte, J Gill, D Dunn, D Pillay, K Porter

**Affiliations:** 1 Clinical Trials Unit - Medical Research Council, London, UK; 2 Robert Koch-Institute, Berlin, Germany; 3 Ulleval University Hospital, Oslo, Norway; 4 Université Bordeaux 2, Bordeaux;; 5 San Paolo Hospital, University of Milan, Milan, Italy; 6 South Alberta HIV clinic, Calgary, Canada;; 7 UCL Medical School, London, UK

## Background

The development of drug resistance is often cited as a disadvantage of early initiation of cART. However, little is known about the long-term probability of detecting drug resistance in individuals initiating cART early.

## Methods

We followed-up patients in CASCADE with well-estimated dates of HIV seroconversion from cART initiated within 1 year of the first HIV positive test to the earlier of: date of detection of drug resistance (IAS-USA list) or last recorded VL. We included patients with a drug resistance test following VL>1000 c/mL and those with VL always <1000 c/mL. The latter were assumed to have no drug resistance. Median survival from cART initiation to detection of drug-resistance was estimated using Kaplan-Meier methods, and log-rank tests were used to explore the association between detection of drug resistance and sex, risk group, cART class, as well as age, calendar year and CD4 at cART initiation.

## Results

Of 609 included patients, median (IQR) age 34 (29,42) years and CD4 count of 364 (243,517) cells/mm3 at cART initiation, 151 had a drug resistance test before cART initiation of whom 7 (4.6%) had transmitted drug resistance (TDR). 29% interrupted treatment for ≥15 days after a median of 0.86 (0.39,1.77) years. 67% and 26% initiated PI and NNRTI-containing cART, respectively. Among 122 patients with at least one resistance test, drug resistance mutation was detected in 19 with during 2392 py follow-up (8/1000 py). Among patients who were detected with a drug resistance mutation, 2 had TDR. The cumulative risk of drug resistance detection was 3% and 7% at 4 and 8 years after cART initiation, respectively. While there was some evidence of effect of CD4 at cART initiation (p=0.043) with higher CD4 being associated with a decreased risk of drug resistance detection, we found no significant association with the other risk factors.

## Conclusions

Although one third of our patients interrupted cART, detection rate of resistance was remarkably low compared to those reported in individuals initiating cART in chronic infection. Our data do not support early cART initiation being associated with long-term probability of drug resistance detection.

